# The Enteropathogenic *E. coli* Effector EspF Targets and Disrupts the Nucleolus by a Process Regulated by Mitochondrial Dysfunction

**DOI:** 10.1371/journal.ppat.1000961

**Published:** 2010-06-24

**Authors:** Paul Dean, Jon A. Scott, Andrew A. Knox, Sabine Quitard, Nicholas J. Watkins, Brendan Kenny

**Affiliations:** Institute for Cell and Molecular Biosciences, Medical School, University of Newcastle, Newcastle upon Tyne, United Kingdom; Institut Pasteur, France

## Abstract

The nucleolus is a multifunctional structure within the nucleus of eukaryotic cells and is the primary site of ribosome biogenesis. Almost all viruses target and disrupt the nucleolus—a feature exclusive to this pathogen group. Here, using a combination of bio-imaging, genetic and biochemical analyses, we demonstrate that the enteropathogenic *E. coli* (EPEC) effector protein EspF specifically targets the nucleolus and disrupts a subset of nucleolar factors. Driven by a defined N-terminal nucleolar targeting domain, EspF causes the complete loss from the nucleolus of nucleolin, the most abundant nucleolar protein. We also show that other bacterial species disrupt the nucleolus, dependent on their ability to deliver effector proteins into the host cell. Moreover, we uncover a novel regulatory mechanism whereby nucleolar targeting by EspF is strictly controlled by EPEC's manipulation of host mitochondria. Collectively, this work reveals that the nucleolus may be a common feature of bacterial pathogenesis and demonstrates that a bacterial pathogen has evolved a highly sophisticated mechanism to enable spatio-temporal control over its virulence proteins.

## Author Summary

Many of the world's most important diseases are caused by bacterial pathogens that deliver effector proteins into the cells of their host. Effector proteins are collectively responsible for causing disease and an important area of research is to define the functions of these proteins and identify how they are regulated once inside the host cell. Here, we show that EspF, a well-studied effector of enteropathogenic *E. coli*, targets the host's nucleolus in both infected and transfected cells and causes extensive nucleolar changes. Previously, only viruses were known to target and disrupt the nucleolus but we show that bacteria other than *E. coli* also disrupt this organelle. Our work also uncovered a novel regulatory mechanism whereby *E. coli* utilises the host mitochondrion to control the extent and timing of EspF nucleolar targeting, ensuring it is strictly a late event in infection. This is the first example of a host organelle controlling the functions of a bacterial effector protein. Taken together, this work reveals a new cellular target for bacterial pathogens and a novel mechanism to regulate the spatio-temporal activity of bacterial effector proteins.

## Introduction

Central to the pathogenesis of many viral pathogens is the requirement to target the nucleolus [1], a sub-nuclear structure found in all eukaryotic cells that is the primary site of ribosome biogenesis. Although the main function of the nucleolus is the synthesis of ribosomes, it is a highly dynamic and multifunctional organelle with a proteome of over 4,500 proteins [2] and has many cell biological functions (reviewed in [3]). The dense concentration of interacting proteins and nucleic acids is crucial to nucleolar function, which if disrupted, can have serious consequences to the cell, leading to disease [3]. One of the best-studied and most abundant nucleolar proteins is nucleolin, an RNA-binding phosphoprotein that represents up to 10% of total nucleolar protein [4] and is crucial for rRNA processing. Although nucleolin is primarily confined to the nucleolus, it is a multifunctional protein, able to shuttle between the nucleus and the cytoplasm and plays important roles in the pathogenesis of many viruses including HIV, poliovirus and hepatitis C [1].

The specific targeting of proteins to the nucleolus is a well-established viral infection strategy exhibited by almost all viral pathogens [1]. Indeed, for several decades viruses have been reported to subvert or hijack specific nucleolar proteins by causing their relocalisation from the nucleolus to another subcellular site such as the cytoplasm where they are presumably unable to perform their nucleolar functions [1]. Unlike their viral counterparts, no other pathogen group including fungi, protozoa or bacteria are known to target or disrupt the nucleolus, presumably reflecting the viral dependence on the host transcription or translation machinery. Many notorious animal and plant pathogenic bacteria that cause some of our most devastating diseases, possess type three- or type four secretion systems to deliver multiple effector proteins directly into eukaryotic cells - a process that is essential to cause disease [5]. These effectors exhibit diverse biochemical activities, subverting many important aspects of host cell physiology and are often highly multifunctional [6], [7]. An emerging theme is functional redundancy between co-delivered effector proteins and therefore it is often difficult to determine the role of individual effectors in disease. A successful approach in understanding the roles of effectors has been to identify effector families or common host cell targets that may be important across a wide range of bacterial pathogens.

Enteropathogenic *E. coli* (EPEC) is a bacterial pathogen that delivers multiple effector proteins into host cells and targets the human small intestine causing severe watery diarrhea with high infant mortality [8]. Unlike related bacterial species such as *Salmonella*, EPEC is non-invasive and from an extracellular position delivers its effectors [7], of which three - Tir, Map and EspF are the best studied [7]. Tir inserts into the host plasma membrane to act as a receptor for the outer membrane protein Intimin [9], mediating intimate bacterial attachment to the host cell. Tir-Intimin interaction also initiates actin-polymerisation to form an actin-rich ‘pedestal’ beneath the bacterium. Map and EspF are highly multifunctional with many overlapping functions as both target mitochondria [10], [11], disrupt tight junctions [12], [13], efface microvilli and inhibit the water transporter SGLT-1 [14] with EspF, but not Map, inhibiting phagocytosis [15]. Although many functions of EPEC effectors have been identified, we know little about their subcellular behaviour or how they are regulated within host cells.

Here, we present the first example of a bacterial protein that specifically targets and disrupts the nucleolus. The EPEC effector EspF is shown to target the nucleolus late in infection where it disrupts a subset of nucleolar factors that are essential for ribosomal biogenesis. We further uncover a novel regulatory mechanism whereby nucleolar targeting by EspF is temporally controlled by EPEC's exploitation of mitochondrial function which is the first example of a host organelle regulating the activities of a bacterial effector. Finally, we demonstrate that other important bacterial species disrupt the nucleolus dependent on effector protein delivery, suggesting that the nucleolus is a common bacterial target.

## Results

### The EPEC effector protein EspF targets the nucleolus

**Figure 1:**
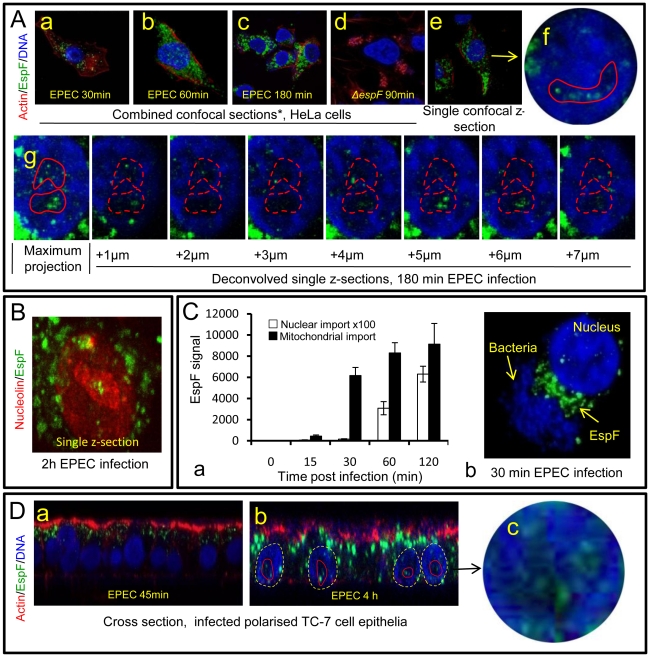
The EPEC effector protein EspF targets the nucleolus late in infection. (A) Immunofluroescence of HeLa cells infected with EPEC or EspF-deficient (*espF*) strain at indicated time points. Images (a–d) show combined confocal z-sections while image (e) shows a single confocal z-section through an infected cell. (f) Punctate EspF within non-DAPI stained nucleolar region (red outline). (g) Confocal z-series through the nucleus of an infected cell - red line encloses the non-DAPI stained nucleolar region. (B) Single confocal z-section through an EPEC-infected cell (2 h) stained for nucleolin and EspF. (C)(a) Quantification of EspF signal in cellular compartments. Units are based on fluorescence intensity; results show mean ± SE (n = 3) with approx. 60 cells per experiment. (b) EPEC-infected cell showing EspF staining concentrated beneath attached bacteria. (D) Single confocal cross-sections of intestinal epithelia infected for 45 min (a) or 4 h (b) with EPEC. The image given in (c) is a magnification of the intestinal TC7 cell nucleus (arrow) showing EspF staining.

**Figure 2:**
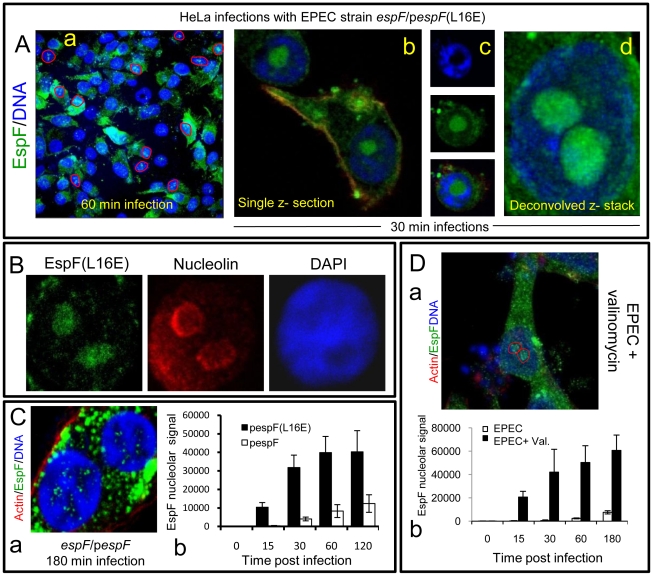
EspF nucleolar targeting is regulated by mitochondrial activity. (A) HeLa cells infected with the EPEC EspF-deficient strain (*espF*) carrying a plasmid expressing the EspF(L16E) variant with a (a) Confocal z-stack showing EspF(L16E) in the cytoplasm and indicated nuclei (red outline) (b) Single confocal z-section through an infected cell showing cytoplasmic and nucleolar staining. (c) Localisation of EspF in the non-DAPI stained nuclear region. (d) Composite deconvolved confocal image of an infected nucleus showing EspF(L16E) throughout the nucleolar region. (B) Confocal section of a HeLa cell infected as in (A) revealing EspF(L16E) colocalises with nucleolin in the nucleolus. (C)(a) Single confocal section of HeLa cells infected with the *espF* strain expressing native EspF from a plasmid. (b) Comparison of nucleolar levels of EspF and EspF(L16E) during infection. Units refer to arbitrary fluorescence signal; results show mean ± SE (n = 3); p<0.01 for all time points compared to t = 0. (D)(a) Confocal section of HeLa cells treated with valinomycin (1 µM) for 2 h prior to EPEC infection showing an increase of EspF in the cytoplasm and nucleolar regions (red outline). (b) Quantification of nucleolar EspF signal from 50 random cells during infection (results show mean ±SE, n = 3; p<0.01 for all time points compared to t = 0).

Previous studies have revealed that EPEC EspF targets mitochondria in infected host cells [10], [16]. Given its many reported functions, we predicted that EspF would target multiple sites in host cells and examined the subcellular location of this effector during early, mid- and late-stage infection. Consistent with previous reports, microscopy of infected HeLa cells revealed an early accumulation (within 30 min) of EspF within punctate cytoplasmic structures (Figure 1Aa-c) that were verified to be mitochondria (Figure S1A). No other cytoplasmic organelle was visibly targeted by EspF during infection (Figure S1A). Mitochondrial targeting by EspF increased up to 60 min post-infection, after which no visible increase in mitochondrial EspF was evident (Figure 1 Aa-c). However, to our surprise, z-axis confocal sectioning (see Materials and Methods) through late-stage (>60 min) infected host cells revealed an unexpected punctate localisation of EspF in the nucleus, within compartments 2–6 µm in size that stained poorly with the DNA dye DAPI (Figure 1Ae-g) – both characteristics of the nucleolus. Parallel studies using cells infected with an EspF-deficient mutant (*espF*) confirmed the specificity of EspF staining (Figure 1Ad). EspF accumulation in the nucleolus was verified by co-staining for nucleolar markers such as nucleolin (Figure 1B; also see Figure 2B), fibrillarin and BMS1 (data not shown). Other EPEC effectors such as Map (Figure S1B) (which has a similar molecular size and similar functions as EspF [7]) and Tir (data not shown) did not accumulate in the nucleus or nucleolus at any time during infection. Thus, EspF specifically targets the nucleolus and is the first example of a bacterially-encoded protein to localise to this sub-nuclear structure.

Quantification of the EspF signal revealed that nucleolar targeting was strictly a late event in infection. Thus, EspF rapidly associated with the mitochondria within 5–15 min of bacterial attachment (see Figure 1Ca) and by 30 min, cells exhibited a marked asymmetry of EspF staining in favour of mitochondria near to the bacterial attachment site (Figure 1Cb), with little, if any, EspF in the nucleus (Figure 1Ca&b; p<0.001). However, by 60 min, the mitochondrial EspF signal began to plateau with no significant increase thereafter (p = 0.21). Following the plateau, EspF became more prominent within the nucleolus - with a ∼40 fold increase between 30 and 120 min (Figure 1Ca). The *in vivo* relevance of this finding was supported by EPEC infection of TC-7 polarised intestinal cells (which represent the natural site of EPEC infection) with EspF targeting the mitochondria of intestinal cells at early time points (Figure 1Da) and nucleolar accumulation consistently a later event (Figure 1Db-c).

### Nucleolar targeting by EspF is temporally regulated by mitochondria

During infection, EPEC progressively causes the dissipation of mitochondrial membrane potential - upon which the mitochondrial import of EspF relies [11], [16]. We therefore hypothesised that EPEC was exploiting mitochondrial import activity to regulate the location of EspF to ensure nucleolar targeting is a late event. To test this hypothesis, we took advantage of an EspF(L16E) variant that cannot target mitochondria [16] and examined its subcellular behaviour. HeLa cells were infected with an EspF-deficient mutant expressing either plasmid-encoded EspF or EspF(L16E) and the cells were stained to examine EspF's location. EspF(L16E) was mainly cytoplasmic (Figure 2Aa) but also targeted the nucleolus much stronger than expected (Figure 2Aa-d), filling the entire nucleolar (non-DAPI stained) region (Figure 2Ac&d). By contrast, the nucleolar staining pattern seen with chromosomal- (see Figure 1) or plasmid-encoded native EspF (Figure 2Ca) was much weaker and more punctate. The nucleolar marker nucleolin confirmed that EspF(L16E) did indeed target the nucleolus (Figure 2B).

Quantification of the EspF signal in infected HeLa cells revealed that the L16E mutation resulted in a significantly more rapid nucleolar signal which was much stronger (at least ∼25 fold; p<0.0001) than native EspF (Figure 2Cb). Indeed, by as early as 15 min. post-infection, the levels of EspF(L16E) in the nucleolus were similar (p = 0.11) to that of native EspF at 120 min, demonstrating that in the absence of mitochondrial targeting, nucleolar uptake occurs almost 8 times faster. This suggested that the late nucleolar targeting of native EspF during infection was directly regulated by mitochondria activity. To further test this prediction, we chemically inhibited mitochondrial membrane potential (MMP) with valinomycin prior to EPEC infection to prevent the mitochondrial import of native EspF. As expected, EspF in these cells was mainly cytoplasmic (Figure 2Da) similar to the L16E variant but also targeted the nucleolus significantly more rapidly and stronger (∼75 fold increase; p<0.0001) compared to untreated EPEC-infected cells (Figure 2Db). These data support the hypothesis that the dissipation of mitochondrial membrane potential induced by EPEC during infection dictates when EspF is available to target the nucleolus and represents a novel mechanism for regulating the cellular location and subsequent function of effector proteins. It is also the first example of a host organelle controlling the function and location of a bacterial effector protein within infected host cells.

### EspF induces extensive redistribution of nucleolin into the cytoplasm

**Figure 3:**
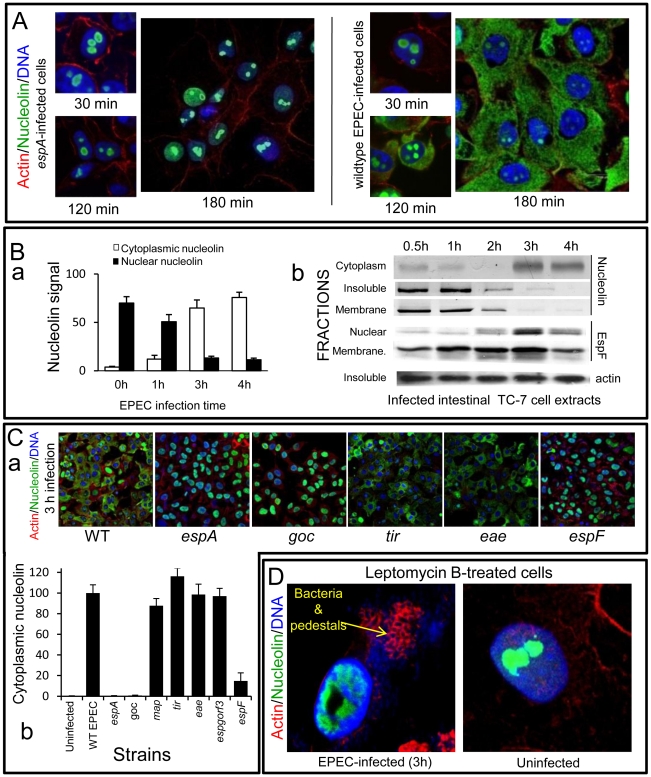
EPEC causes extensive redistribution of nucleolin dependent on EspF. (A) Immunofluorescence of HeLa cells infected with EPEC or the effector delivery-defective strain *espA* (B)(a) Quantification of nucleolin signal in the cytoplasm and nucleus of HeLa cells infected with EPEC. Results show mean ±SE (n = 3). (b) Western blots of EPEC-infected intestinal TC-7 cell fractions probed for nucleolin, EspF or actin. Insoluble fraction contains nucleus plus bacteria (C)(a) Immunofluorescence of HeLa cells infected with EPEC strains: WT (wild type EPEC), *espA* (effector delivery defective), *espF*, *tir*, *eae* (Intimin), *goc* (deficient for delivery of at least 11 effectors, including EspF). (b) Cytoplasmic nucleolin signal in host cells that were infected with effector-deficient EPEC strains was quantified over 10 fields of view per experiment (bars show mean ± SE, n = 3). (D) HeLa cells treated with an inhibitor of nuclear export (leptomycin B; 5 ng/mL) for 2 h prior to 3 h EPEC infection and stained for nucleolin. Arrow indicates actin pedestals mediated by EPEC effectors.

To investigate a specific function for nucleolar targeting by EspF, we assessed changes in important nucleolar components. As nucleolin is the most abundant nucleolar protein, essential for ribosomal biogenesis, it was the primary focus. Microscopy of uninfected HeLa cells stained with nucleolin antibodies revealed nucleolin was exclusively found within the nucleus/nucleolus (not shown), which remained unchanged following a 30 min EPEC infection (Figure 3A). However, at later infection times (>120 min), EPEC caused a dramatic relocalisation of nucleolin from the nucleolus into the cytoplasm (Figure 3A), which by 180 min was almost exclusively cytoplasmic. Quantification of the nucleolin signal in the cytoplasm and nucleus revealed an inverse relationship during EPEC infection (Figure 3Ba), suggesting the cytoplasmic nucleolin arose directly from the nuclear pool. Importantly, the *espA* EPEC mutant (which cannot deliver effectors into host cells) did not induce any visible changes of nucleolin (Figure 3A) suggesting that nucleolin relocalisation was indeed mediated by effector proteins.

Similar results were obtained using polarised intestinal TC-7 cells (Figure S2Aa) and supported by Western blot analysis which showed that at late infection, nucleolin levels decreased in the ‘insoluble’ (nuclei-containing) and membrane fractions with a corresponding increase in the cytoplasmic fraction (Figure 3Bb). Both events coincided with increased EspF in the nuclear fraction between 2–4 h post-infection (Figure 3Bb) while the EspF signal in the ‘membrane’ fraction (containing mitochondria) increased gradually from 30 min up to 3 h (Figure 3Bb) – consistent with the previous data.

To determine which EPEC effector was causing the relocalisation of nucleolin, we infected HeLa cells for 3 h with various EPEC strains lacking effector genes and stained for nucleolin (Figure 3C). This revealed a central role for EspF in nucleolin relocalisation with no role for the effectors EspG, Orf3, Map, Tir or the outer membrane protein Intimin/*eae*. The finding that the *espF* mutant carrying the EspF L16E variant on a plasmid induced greater cytoplasmic nucleolin than native plasmid-encoded EspF supported the idea that nucleolar targeting may be involved (Figure S2Ab) Quantification of nucleolin levels in infected cells did reveal a minor but significant (p<0.001) increase in cytoplasmic nucleolin in Δ*espF*-infected cells (Figure 3Cb) compared to cells infected with the *espA* mutant, suggesting a lesser role for other effector(s) in the process. These effectors are evidently missing from the multiple knockout mutant *espGorf3Δcore* (goc; Figure 3Cb) that is deficient for delivery of at least 11 EPEC effectors, including EspF [17]. Importantly, the *espF* mutant displayed no significant defects in adherence or effector-mediated actin-pedestal formation compared with wildtype EPEC (Figure S2B).

Nucleolin can shuttle between the nucleus and cytoplasm [4] and EspF may alter its equilibrium in favour of cytoplasmic accumulation. To test this hypothesis, cells were pre-treated with leptomycin B (LMB) to inhibit nuclear export of proteins prior to EPEC infection. Although this treatment had no effect on nucleolin location in uninfected cells and did not interfere with EPEC effector-driven actin rearrangements (Figure 3D), it abolished any detectable EPEC-mediated mobilisation of nucleolin into the cytoplasm (Figure 3D). However, LMB treatment failed to prevent EPEC-mediated nucleolin mobilisation from the nucleolus into the nucleus (Figure 3D and Figure S2C), suggesting EspF specifically induces the loss of nucleolin from the nucleolus which is then mobilised into the cytoplasm via classical (LMB-sensitive) nuclear export.

### EPEC disrupts a subset of nucleolar factors essential for ribosomal biogenesis

**Figure 4:**
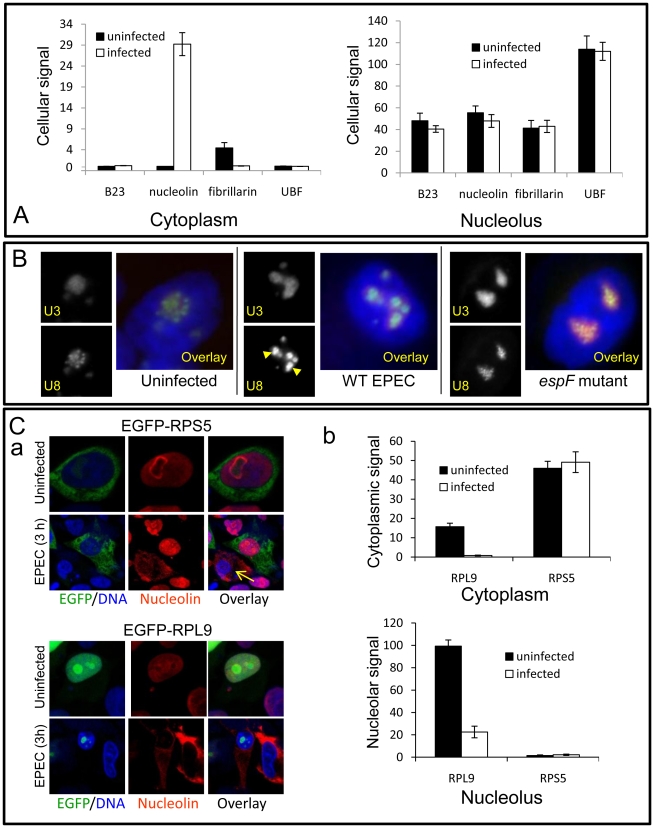
EPEC disrupts a subset of nucleolar factors. (A) Quantification of EGFP-tagged nucleolar proteins in the cytoplasm or nucleolus of uninfected or EPEC-infected (3 h) HeLa cells; bars shows mean ± SE, n = 3. There was no significant differences for any construct before or after infection (p>0.3) except for increased cytoplasmic nucleolin (p<0.0001) and decreased fibrillarin (p = 0.002). (B) Epifluorescence of U3 and U8 snoRNA antisense probes in HeLa cells infected for 3 h with wildtype (WT) EPEC or the EspF-deficient (*espF*) strain. Images show infected cell nuclei. Arrowheads indicate the condensation of U8 snoRNA in wildtype EPEC infected cells. (C) (a) Confocal image of EGFP-RPS5 and EGFP-RPL9 expressed in HeLa cells before and after a 3 h EPEC infection. Arrow shows a non-transfected cell stained with nucleolin. (b) Quantification of EGFP levels in the nucleolus and cytoplasm of transfected cells from (C) (a); bars show mean ± SE, n = 3.

To determine whether nucleolin mobilisation into the cytoplasm was a specific EspF-mediated event, several nucleolar proteins were assessed by immuno-detection or tagging with EGFP. The location of EGFP-tagged B23 (a nucleo-cytoplasmic shuttling protein), upstream binding factor (UBF, a nucleolar transcription factor) or fibrillarin (found in the dense fibrillar component (DFC) of the nucleolus) remained unchanged after a 3 h EPEC infection (Figure 4A) - despite extensive nucleolin redistribution in the same cells (Figure S3A). This was supported by immunostaining for the nucleolar proteins fibrillarin and BMS-1, which remained unaltered following infection (Figure S3B). Of the nucleolar proteins tested, only EGFP-nucleolin entered the cytoplasm following a 3 h EPEC infection (Figure 4A and Figure S3A), revealing that the redistribution of nucleolin into the cytoplasm is a specific event. Surprisingly, we did not detect a significant loss of EGFP-nucleolin from the nucleolus during infection (Figure 4A) unlike that seen with native nucleolin (Figure 3B) suggesting that the N-terminal EGFP tag or the high level of expression of EGFP-nucleolin may affect the mobilisation of this protein.

Given nucleolin's essential role in ribosome biogenesis, we examined whether other ribosome-associated factors were altered by EPEC infection. Small nucleolar RNAs (snoRNAs) U8 (located in the nucleolar DFC) and U3 (located in DFC and granular component of the nucleolus) are essential for ribosomal biogenesis [18], [19]. *In situ* hybridisation for U8 and U3 snoRNA in uninfected cells revealed particulate and diffuse nucleolar staining patterns respectively (Figure 4B), consistent with previous reports [20]. Infection with EPEC for 1 h had no detectable effect on U3 or U8 staining pattern (Figure S3D) but after a 3 h infection the particulate U8 signal strongly coalesced while U3 remained unaltered (Figure 4B). This effect on U8 was not induced by the *espA*- (effector-delivery defective strain) or the EspF-deficient mutant (*espF*) (Figure 4B), revealing that EspF was responsible for the change in U8 snoRNA. By contrast, EPEC did not alter the distribution of native fibrillarin (Figures S3A&B), which is associated with U8 in the DFC [19], implying that the EspF-induced alteration of U8 distribution is a highly specific event.

The importance of U8 snoRNA and nucleolin in ribosomal biogenesis further led us to examine the levels and distribution of ribosomal proteins RPL9 and RPS5. In agreement with previous work [21], EGFP-RPL9 was detected in the nucleus along with a weak cytoplasmic localisation (Figure 4C). EPEC infection significantly reduced the total amount of EGFP-RPL9 in both compartments (Figure 4Ca&b; p = 0.002 in both cases). This was supported by Western blot of native RPL9 which was reduced by wild type EPEC infection, dependent on EspF (Figure S3C) By contrast, EGFP-RPS5 was mainly cytoplasmic and remained unaffected by EPEC infection (Figure 4Ca&b; p = 0.7) suggesting that EPEC alters the levels of specific ribosomal proteins. Unexpectedly, although EPEC did not affect RPS5 levels, expression of EGFP-RPS5 completely prevented the EPEC-mediated mobilisation of nucleolin, an event that was evident in neighbouring non-transfected (EGFP-RPS5 negative) cells (Figure 4Ca; arrow). This inhibitory effect of EGFP-RPS5 in preventing nucleolin mobilisation was supported by quantification (Figure S3E), suggesting that either directly or indirectly, this specific ribosomal protein is able to interfere with EspF-mediated nucleolin redistribution. Overall, these results show that EPEC alters the distribution and/or levels of a specific subset of nucleolar proteins (nucleolin and not fibrillarin, B23, UBF and BMS1), small nucleolar RNAs (U8 and not U3) and ribosomal proteins (RPL9 and not RPS5). As these factors are all essential for ribosomal biogenesis, this supports the notion that ribosome biosynthesis may be specifically disrupted by EPEC. Preliminary Northern blot data also indicates that transcription of pre-rRNA and downstream rRNA cleavage events (data not shown) are also disrupted by EspF when expressed in host cells, dependent on a defined nucleolar targeting domain (as described below).

### EspF alone mediates nucleolar targeting via an N-terminal domain to cause nucleolin relocalisation

**Figure 5:**
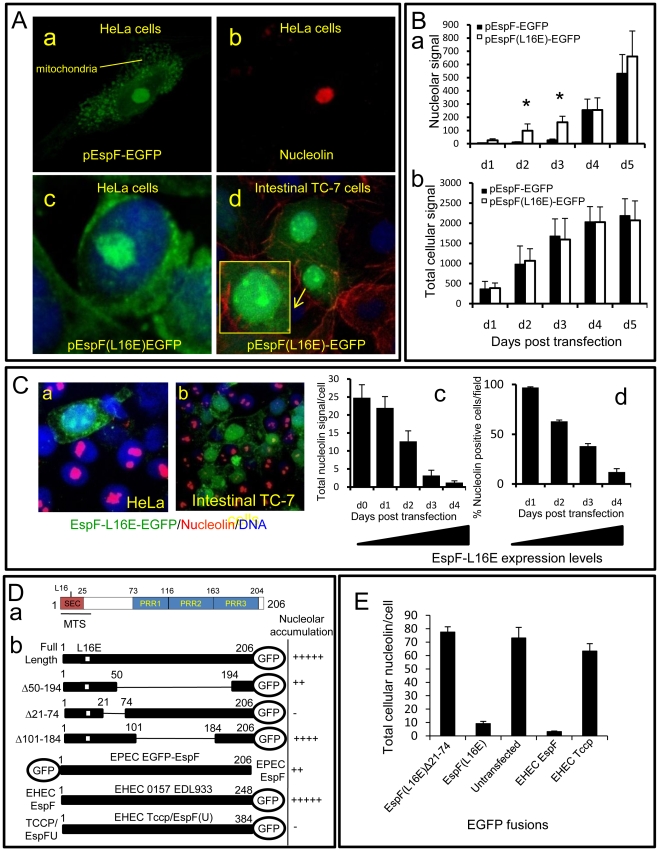
The N-terminal domain of EspF mediates nucleolar targeting and loss of nucleolin. (A) Confocal images of HeLa (a–c) and intestinal TC-7 cells (d) expressing EspF-EGFP or EspF(L16E)-EGFP. Inset in d) shows a contrast-enhanced magnified nucleus. (B) Quantification of the (a) nuclear and (b) total cellular EGFP signal from HeLa cells transfected with EGFP-tagged EspF or EspF(L16E). Units represent arbitrary fluorescent signal (mean ± SE, n = 3, 10 random cells per experiment). Asterisks indicate significant differences between the EspF variants (p<0.01) at corresponding time points. (C) Expression of EspF(L16E)-EGFP in (a) HeLa or (b) polarised intestinal TC-7 cells and stained for nucleolin. (c) Quantification of total levels of nucleolin in HeLa cells expressing EspF(L16E)-EGFP. (mean ± SE, 10 random transfected cells, n = 3) (d) Percentage of HeLa cells exhibiting nucleolin signal at or above background levels following transfection with EspF(L16E)-EGFP (mean ± SE, 10 random transfected cells, n = 3). (D) (a) EspF protein sequence indicating the N-terminal secretion (SEC) domain, three C-terminal polyproline repeats (PPR), a mitochondrial targeting sequence (MTS) and residue L16E critical for mitochondrial targeting. (b) Nucleolar accumulation of EspF(L16E) constructs and EHEC EspF homologues (EspF and Tccp) fused to EGFP after 1–5 day expression in host cells. Nucleolar score was based on EGFP intensity in the nucleolar region with native EspF given (+++++) and EGFP alone given (−) (n = 4 with 5 fields assessed per experiment). (E) Nucleolin signal in cells transfected with EspF variants quantified as described in (C).

To determine whether EspF alone is sufficient to target the nucleolus and mediate the redistribution of nucleolin, we expressed EspF (and its L16E variant) as an EGFP fusion protein within host cells. Microscopy revealed EGFP alone (data not shown) or EspF(L16E)-EGFP were predominantly cytoplasmic, while native EspF-EGFP targeted the mitochondria (Figure 5Aa-c). In addition, both EspF fusion variants strongly targeted the nucleolus of polarised (TC-7) and non-polarised (HeLa) cell types (Figure 5Ad and Figure S4A) revealing EspF alone is sufficient to target this organelle. Quantification of the EGFP cellular signal in HeLa cells revealed that EspF(L16E)-EGFP was present within the nucleolus 2–3 days before EspF-EGFP (Figure 5Ba), despite no significant differences in total expression levels (Figure 5Bb; p>0.8). The delay for native EspF to accumulate in the nucleolus, compared with the L16E variant, further supports the idea that mitochondrial import regulates EspF-nucleolar targeting, as shown with the infection data.

Host cells that were transfected with EspF unexpectedly displayed a complete loss of nucleolin in all cellular compartments in both non-polarised and polarised cells (Figure 5Ca&b). EspF(L16E)-EGFP induced a more rapid loss of nucleolin than EspF-EGFP (data not shown), presumably due to its more rapid accumulation in the nucleolus. Quantification of nucleolin within EspF(L16E)-EGFP-transfected cells revealed that nucleolin gradually diminished ∼25-fold to near background levels by day 4 post-transfection (Figure 5 Cc&d) correlating with increasing EspF(L16E)-EGFP expression (Figure S4B). Indeed, after 4 days, most cells expressing EspF(L16E)-EGFP (∼88%) exhibited no detectable nucleolin above background levels (Figure 5Cd) while cells transfected with control or empty EGFP vectors displayed normal nucleolin levels (Figure S4C). The complete absence of nucleolin in cells transfected with EspF, in contrast to EPEC-infected cells, possibly reflects incubation time differences (i.e. hours vs. days respectively), levels of EspF or a role for additional EPEC factors.

**Figure 6:**
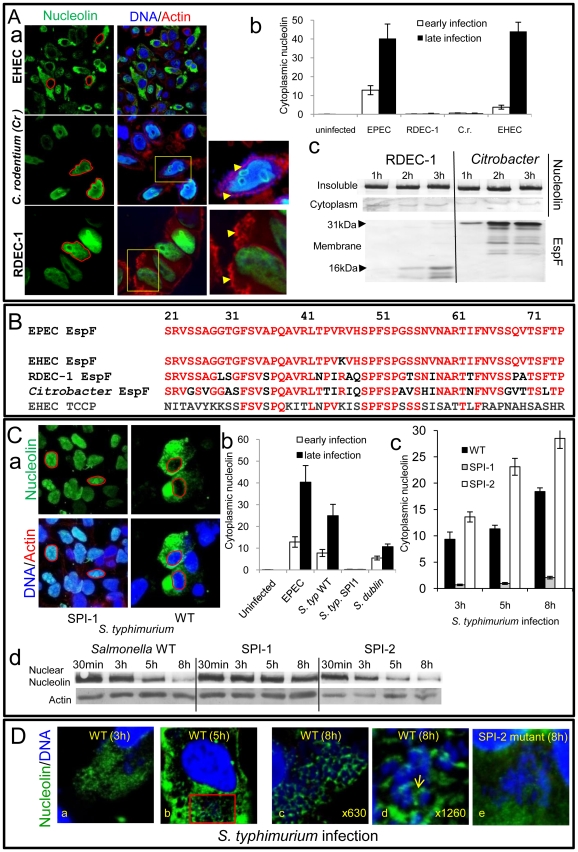
Nucleolin is mobilised by other bacterial pathogens dependent on effector delivery. (A) Nucleolin location within host cells following infection with EPEC-related strains that infect humans (EHEC), rabbits (RDEC-1) or mice (*Citrobacter rodentium*; C.r.). (a) Immunofluorescence of infected cells after a 3 h (EHEC), 15 h (C.r.) or 8 h (RDEC-1) infection. Red line indicates nuclear perimeter. Yellow boxes are enlarged to show effector-driven pedestal formation (yellow arrow heads) by RDEC-1 and *Citrobacter* respectively – demonstrating that effector delivery is not compromised in these strains (b) Quantification of cytoplasmic nucleolin levels in cells infected with indicated bacterial pathogens (mean ± SE, n = 3 independent experiments). (c) Western blot showing changes in nucleolin levels in cytoplasmic and nuclear (insoluble) fractions after infection with *Citrobacter* and RDEC-1 for the indicated time points. Host cell delivery of EspF (16 kDa and 31 kDa respectively) by these strains is also shown and is quantified in Figure S5C. (B) EPEC EspF sequence 21–74 (putative nucleolar targeting region) and corresponding regions of EspF homologues from related pathogens; conserved residues in red. (C) (a) Immunofluorescence of infected cells after 8 h infection with WT (wildtype) *Salmonella typhimurium* or a *Salmonella* strain (SPI-1) unable to deliver effectors through the SPI-1 system. Red line indicates nuclear perimeter. (b) Quantification of cytoplasmic nucleolin after 3 h and 5 h EPEC infection or 3 h and 8 h infections with *S. typhimurium* (*S. typ* WT or *S. typ* SPI-1) or *Salmonella dublin* (mean ±SE for 3 separate experiments). (c) Quantification of cytoplasmic nucleolin in cells infected with *S. typhimurium* strains (mean ± SE for 3 separate experiments). (d) Representative Western blot of nucleolin in the insoluble (nuclear-containing) fraction of host cells infected for the indicated times with the *Salmonella* strains given. (D) Representative confocal z-sections of cells infected with wild type *S. typhimurium* (a–d) or the SPI-2 mutant (e) with the latter having a clear defect in recruiting nucleolin. The red box in (b) is magnified in Figure S5E.

Despite the differences in nucleolin fate, the transfection system provided a convenient means to screen for features of EspF that are required for nucleolar targeting and/or nucleolin loss. Bioinformatic analysis of the 206 residue sequence of EspF failed to identify a putative nuclear localisation signal (NLS; see Materials and Methods) while no consensus nucleolar localisation signal (NoLS) is known at present [1]. We therefore investigated the ability of EspF variants carrying internal deletions to accumulate within the nucleolus. Deletion of EspF residues 50–194, which removes three polyproline repeats (PRR) that make up the majority of this protein (Figure 5Da), only partially impaired nucleolar accumulation (Figure 5Db) while deletion of residues 101–184 displayed no visible defect in nucleolar targeting, ruling out a role for the polyproline repeats (Figure 5Db). The residual ability of EspF Δ50–194 to accumulate in the nucleolus suggested that the remaining N- or C-terminal regions were important. N-terminal EGFP fusions were also defective in nucleolar targeting (Figure 5Db) implicating the N-terminal EspF region. Indeed, deletion of region 21–74 completely abolished EspF accumulation in the nucleolus (Figure 5Db). However, like EGFP alone, the EspF(Δ21–74) variant was able to enter the nucleus (Figure S4D) – thus revealing a specific role for residues 21–74 in targeting EspF to the nucleolus. Interestingly, the closely related EPEC pathogen, enterohemorrhagic *E. coli* (EHEC 0157:H7) encodes two EspF homologues – EspF and EspFU/Tccp (herein Tccp) which differ greatly in the N-terminal (21–74) region (98% vs. 32% identity respectively; see Figure 6B). Indeed, whereas EHEC EspF-EGFP targeted the nucleolus, Tccp-EGFP did not (Figure 5Db), further supporting a role for region 21–74 in nucleolar targeting by EspF.

The identification of a putative nucleolar targeting region enabled us to determine whether EspF disruption of nucleolin was specifically linked to EspF nucleolar targeting. Thus, EspF(L16E)Δ21–74 fused to EGFP was expressed in mammalian cells and nucleolin levels were quantified revealing that unlike full length EspF (Figure 5E), the Δ21–74 variant had no effect on nucleolin levels relative to untransfected cells (Figure 5E; p = 0.83). Furthermore, the EHEC EspF homologue also caused extensive nucleolin loss (Figure 5E) while the Tccp homologue was similar to untransfected cells (Figure 5E; p = 0.55). Taken together, these data demonstrate that the ability of EspF to target the nucleolus is directly linked with the loss of nucleolin.

### The nucleolus – a common target of bacterial pathogens?

To investigate whether the nucleolus is targeted by other bacterial species that deliver effectors into host cells, we initially examined the ability of other EspF-encoding pathogens to induce nucleolin redistribution. Interestingly, while the human-specific pathogen enterohemorrhagic *E. coli* (EHEC) induced nucleolin relocation into the cytoplasm of HeLa cells, two other closely-related strains RDEC-1 (rabbit-specific EPEC) and *Citrobacter rodentium* (mouse-specific; Cr) did not (Figure 6Aa&b) even after very long infection times. This inability was not linked to effector delivery defects as both Cr and RDEC-1 triggered extensive effector-mediated actin-pedestal formation (Figure 6Aa and Figure S5A) and importantly both delivered high levels of EspF into host cells (Figure 6Ac and Figure S5B-C). Interestingly, comparison of the EspF sequences linked to nucleolar targeting (i.e. residues 21–74) revealed 1, 12, 17 and 36 substitutions for EspF of EHEC, RDEC, Cr and EHEC EspFU/Tccp, respectively, compared with EPEC EspF (Figure 6B). Thus, the presence of multiple substitutions in the nucleolar targeting region of EspF likely explains the inability of RDEC and Cr to induce nucleolin redistribution.

Importantly, studies with *Salmonella* species that target humans (*S. typhimurium*) and cattle (*S. dublin*) but do not encode EspF homologues revealed that both species induced extensive mobilisation of nucleolin into the cytoplasm (Figure 6Ca-b). *Salmonella* encode two effector delivery systems, SPI-1 and SPI-2, with the former essential for host cell invasion, while both systems contribute to the formation of *Salmonella*-containing vacuoles (SCV) [22]. Interestingly, a SPI-1 mutant failed to induce nucleolin redistribution (Figure 6Ca-b) in HeLa cells suggesting that effectors delivered by the SPI-1 system are required for this process. By contrast, a SPI-2 mutant that invades host cells and delivers SPI-1 effectors [23] induced significantly greater levels of cytoplasmic nucleolin (p = 0.008) compared with the wild type strain (Figure 6Cc and Figure S5D), suggesting that SPI-2 effector(s) act to attenuate redistribution. Western blot analysis supported the microscopy data as wild type *Salmonella* and the SPI-2 mutant caused a progressive decrease in nuclear nucleolin that was not evident with the SPI-1 mutant (Figure 6Cd). In-depth confocal examination of host cells infected with wildtype *Salmonella* revealed diffuse cytoplasmic nucleolin by 3 h post-infection (Figure 6Da) which strongly sequestered around intracellular SCV by 5–8 h post-infection (Figure 6Db-d and Figure S5E) as supported by the absence of nucleolin in bacterial-free cytoplasmic regions (Figure S5F). Parallel studies with the SPI-2 mutant revealed a major defect in nucleolin sequestration (Figure 6De), suggesting a role for SPI-2 delivered effector(s) in this process. Overall, these findings support the contention that the nucleolus and its major component nucleolin are commonly targeted, not only by viruses, but also by bacterial pathogens.

## Discussion

In this study, we describe the first example of a non-viral pathogen that specifically targets a protein to the nucleolus and also define a novel mechanism for the spatial/temporal control of a bacterial effector protein within host cells. We further demonstrate that bacterial pathogenic species with invasive or non-invasive lifestyles employ their effector delivery systems to disrupt the nucleolus. This work not only reveals a novel effector function and a new eukaryotic target for bacterial effectors, it also shows that bacteria have evolved a highly sophisticated mechanism to control the activities of their virulence proteins by utilising host organelles.

Multiple lines of evidence support the contention that enteropathogenic *E. coli* specifically targets EspF to the nucleolus. Firstly, in EPEC-infected cells, EspF specifically colocalised with nucleolar markers within a distinct nuclear sub-compartment (2–6 um sized DAPI-refractive organelle). Secondly, EspF-EGFP fusions targeted the nucleolar region alone, irrespective of EPEC infection. Thirdly, two other EPEC effectors, Tir and Map, were never detected in the nucleus/nucleolus despite Map sharing many features with EspF [7]. Fourthly, nucleolar targeting by EspF induced specific redistribution of nucleolin (but not B23, fibrillarin, UBF1 or BMS1) into the nucleoplasm from where it entered the cytoplasm via the host's canonical nuclear export pathway. And finally, EspF residues 21–74 were identified as the nucleolar targeting domain required for nucleolar accumulation and mobilisation of nucleolin. The identity of the putative nucleolar targeting domain was supported by the finding that EspF homologues carrying multiple substitutions within this region failed to target the nucleolus and/or trigger nucleolin redistribution, unlike a homologue with a single substitution.

At present, there is little understanding about how bacterial effectors with multiple functions, such as EspF, are regulated during infection. Exceptions include *Salmonella* SopE and SptP whose functions are temporally controlled through host-mediated proteosomal degradation and ubiquitination [24], [25] while *Yersinia* YpkA activation is dependent on host factors [26]. Here, we report a new mechanism of effector regulation involving the activity of a host organelle - the mitochondrion. Thus, during infection, EspF rapidly accumulates in mitochondria – dependent on a functional mitochondrial membrane potential (MMP) [10], [11], [16]. We postulated that the progressive loss in MMP caused by EPEC during infection [11], [16] would regulate when EspF became available for nucleolar targeting. This hypothesis was supported by (i) chemically inhibiting MMP and (ii) abolishing EspF's mitochondrial signal sequence – both of which dramatically increased the speed and intensity of EspF within the nucleolus. Thus, the data suggest that EPEC induces mitochondrial dysfunction to control when EspF is available to target the nucleolus. This manipulation of host mitochondria represents a novel regulatory mechanism to control effector proteins that could potentially be employed by other pathogens that target proteins to this organelle [27].

One obvious question is why does EPEC target EspF to the nucleolus? The late nucleolar targeting of EspF within polarised intestinal epithelia suggests that EspF's nucleolar function is unlikely to be involved in the rapid disease-associated events such as intimate adherence, actin nucleation, microvilli effacement or inhibition of water transporter - all events linked with EspF function [7]. Although EspF nucleolar accumulation correlates temporally with EPEC's disruption of epithelial barrier function, we have found no evidence for a link between the two processes as EspF in an *eae* mutant - which cannot disrupt barrier function [13], targets the nucleolus and causes nucleolin mobilisation, suggesting that EspF nucleolar targeting alone is not linked to tight junction disruption. Intriguingly, we did find that nucleolin is recruited to the EPEC infection site, similar to reports with EHEC [28], but no role for EspF nucleolar targeting could be found in the process (not shown). EspF's role in mediating apoptosis was also not considered to be involved in nucleolar targeting as the L16E EspF variant, which readily targets the nucleolus and causes nucleolin mobilisation, has been documented to not cause apoptosis in host cells [16], [29]. In addition, we find very low levels of apoptosis in HeLa cells infected with the Intimin-deficient EPEC mutant (not shown), despite EspF targeting the nucleolus in this strain.

A likely clue about why EspF targets the nucleolus relates to the extensive EspF-mediated relocation of nucleolin into the cytoplasm and the altered distribution of the U8 small nucleolar RNA (snoRNA) – both essential for ribosome biogenesis. These nucleolar changes were highly specific as other nucleolar proteins (B23, fibrillarin, UBF and BMS1) and U3 snoRNA remained unaltered by EPEC infection. Ribosome biogenesis relies upon the precise co-localisation of specific nucleolar factors within the nucleolus and therefore the complete removal of nucleolin from the nucleolus, along with the marked alteration in U8 snoRNA would undoubtedly have a negative impact on ribosome biogenesis. In line with this, the levels of the ribosomal protein RPL9 (native and the EGFP-tagged variant) were reduced following EPEC infection that was dependent on EspF, while previous proteomic studies on intestinal cells show that the levels of many ribosomal proteins are reduced following EPEC infection [30]. Preliminary data also reveals a blockage during pre-rRNA processing in host cells expressing EspF, which is dependent on EspF's nucleolar targeting domain (data not shown). Future studies will attempt to decipher the mechanism of ribosomal synthesis inhibition and its role in EPEC infection. The reason for targeting ribosomal factors is unclear but shutting down ribosome biogenesis would potentially free up resources for the bacterium as it represents a large proportion of the total energy consumption by host cells [31]. Mammalian ribosomes are very stable (60–120 hr half life) [32], suggesting that inhibition of *de novo* ribosomal biogenesis would not have an immediate impact on protein synthesis but would undoubtedly have greater significance during *in vivo* infections which can last days to weeks [8]. Interestingly, ribosomal proteins also have extra-ribosomal functions in modulating transcriptional factor activity and/or translation of specific mRNAs [33] providing another putative rationale for targeting specific ribosomal proteins.

Like many bacterial effectors, EspF does not play an essential role in disease as *espF*-deficient mutants have only a partial or negligible defects in virulence, at least in the mouse-*Citrobacter* model [34]. This is likely due to effector redundancy as *Citrobacter*, like EPEC, delivers over 20 effector proteins into the host cell that individually only have small effects *in vivo.* Unfortunately, there is no amenable animal model for EPEC and therefore a role for EspF nucleolar targeting in disease has not been possible to ascertain. The finding that RDEC (rabbit-specific) and *Citrobacter* (mouse-specific) do not disrupt the nucleolus/nucleolin during infection suggests that these bacterial species would not be suitable to determine EspF's nucleolar role in disease. Thus, while the role of nucleolar targeting is an intriguing aspect of EspF's function, its role in EPEC disease remains unclear but likely contributes to the overall fitness of the pathogen in the host environment.

In this study, four out of six tested bacterial strains/species that have either invasive (*S. typhimurium* or *S. dublin*) or non-invasive (EPEC and EHEC) life-styles induced an almost complete redistribution of nucleolin from the nucleolus to the cytoplasm, often resulting in no detectable nucleolin within the nucleolus. Nucleolin provides a good indicator of nucleolar subversion because it is the most abundant nucleolar protein and plays an essential role in ribosome biogenesis [4], [35]. The consequences of a complete loss of nucleolin from the nucleolar region are undoubtedly deleterious to the host cell as nucleolin, by inference, could no longer perform its vital nucleolar functions. Further investigations with *Salmonella* showed that two separate effector delivery systems (SPI-1 and SPI-2), which deliver different sets of effectors into the host cell, differentially modulate nucleolin relocation. Thus, the ability of *S*. *typhimurium* to mobilise nucleolin into the cytoplasm was dependent on the SPI-1 system while SPI-2 was required to sequester cytoplasmic nucleolin around intracellular bacteria. This co-cooperativity of two distinct effector-delivery systems in altering the cellular location of nucleolin supports the contention that subversion of nucleolin is a specific virulence-associated event. Collectively, this work suggests that various bacterial pathogens which deliver proteins into the host may also target and manipulate the nucleolus and/or nucleolar proteins.

In conclusion, the involvement of the nucleolus and disruption of nucleolar factors is a new concept in bacterial pathogenesis and the nucleolar field. For decades, the importance and relationship between viruses and the nucleolus has been well established and in light of the work presented here, this relationship should now be extended to bacterial pathogens. Indeed, this work should encourage efforts to determine whether many other important bacterial pathogens target and utilise this sub-nuclear structure. As over 350 bacterial effector proteins have been identified [6] that are delivered into human, animal or plant hosts, it is highly likely that a subset of these proteins behaves like EspF and target the nucleolus. Moreover, with recent proteomic advances in the nucleolar field and the acceptance that the nucleolus is highly dynamic and multi-functional, bacteria will undoubtedly provide an important resource to further our understanding of the nucleolus and its role in health and disease.

Finally, bacterial effectors are intriguing molecules – often highly modular by design and displaying multiple functions. How these proteins are regulated once inside the host cell remains an important question and the work presented here demonstrates the high level of sophistication employed by bacterial pathogens to tightly control their effector proteins. By evolving such regulatory mechanisms, bacterial pathogens ensure the functional repertoire of their virulence proteins are maximised – thereby increasing the bacterium's capacity to subvert cellular processes.

## Materials and Methods

### Cell culture, bacterial strains, plasmids and general procedures

Infection assays, immunofluorescence, Western blot and cell culture methods used in this study have been described elsewhere [13], [14], [36] although a detailed description of these methods are given in Protocol S1. Strains, plasmids, oligonucleotides and reagents are given in Table S1. EspF deletion constructs were made by inverse PCR as described in Protocol S1. *In situ* hybridisation for U8 and U3 was performed as previously described [20]. To assess levels of ribosomal protein L9 in infected TC-7 cells, the cells were infected for 5 h with various EPEC strains and the bacteria were killed by exposure to 100 µg/mL gentamycin for 1 h. Cells were left for an additional 36 h, after which they were lysed with triton X-100 and processed for Western blot as described in Protocol S1. Where cell synchronisation was sought, particularly for quantification analysis, a standard double thymidine block was used by incubating cells in DMEM containing 2 mM thymidine for 19 h, removing the thymidine for 10 h and replenishing 2 mM thymidine for a further 17 h. After this time the thymidine was removed and the synchronised cells were used the following day(s). For transfections, Lipofectamine 2000 was used for all cells types according to the manufacturer's instructions.

### Confocal microscopy and image analysis

Confocal analysis was performed on a Leica TCS SP2UV confocal microscope. Cells were fixed and stained cells on coverslips or membrane filters as previously described [13]. Cells were visualised with a ×63 objective lens by making a series of optical slices through the cell along this z-axis (i.e. parallel to the coverslip). Images were routinely deconvolved using Huygens Professional Deconvolution software with default parameters but with at least 50 iterations. Maximal confocal projections (the entire reconstructed ‘z-stack’) or single z-slices are indicated in Figure legends. Fluorescence intensity was determined using Leica quantification software or Image J (NIH) and presented as arbitrary fluorescence values based on the mean numbers of pixels for each channel. Total fluorescence from individual cells was determined by capturing the cell as a region of interest (ROI) using confocal software. The nuclear/nucleolar signal was measured by making an ROI around these cellular structures while the cytoplasmic signal was determined by subtracting the nuclear signal from the total cell fluorescence. Routinely, negative control slides were used to set base parameters for each series of slides, which was maintained during visualisation, ensuring the detected signal was specific to the fluorophore being examined.

### Statistical analysis and bioinformatics

In all cases, unless otherwise stated, experiments were repeated independently 3 times. Presented graphs represent the mean ± SEM and where confocal microscopy was used for quantification, results represent at least 50 cells for each experiment over 3–5 randomly chosen fields of view unless otherwise stated. Where necessary, comparison of means was performed using the non-parametric Mann-Whitney U test with p values less than 0.01 taken as a significant. Bioinformatic analysis of the EspF protein was performed using BLASTP, BLAST PSI, PredictNLS (http://cubic.bioc.columbia.edu/cgi/var/nair/resonline.pl) and MITOPROT (www.expasy.org).

## Supporting Information

Table S1 Cell lines, Bacterial Strains, Plasmids, and Oligonucleotides
(0.04 MB DOC)

Figure S1 EspF and Map staining in infected HeLa cells. (A) Colocalisation of DsRED-MITO (a mitochondrial marker, red) with EspF (green) in HeLa cells following a 60 min infection with EPEC. (B) Immunofluorescence using HA antibodies to detect MapHA (green) in HeLa cells after a 3 h infection with Δ*map*/*pmap*HA. Actin staining (red) shows pedestals on the cell surface; DNA (blue).
(0.03 MB PDF)

Figure S2 Nucleolin localisation and bacterial binding in host cells infected with EPEC. (A) (a) Quantification of the cytoplasmic nucleolin signal in EPEC infected TC-7 intestinal cells. Cytoplasmic nucleolin levels were counted over 6 fields of view (results show mean ± SE). (b) Quantification of the cytoplasmic nucleolin signal in HeLa cells infected with the indicated EPEC strains. (B) Left Graph: Number of pedestals per infected cell was not significantly different between WT and *espF* mutant after 40 min infection period (10 fields of view counted, results show mean ± SE). Right Graph: The number of bacteria attached to infected host cells after 40 min infection with indicated EPEC strains (results show mean ± SE, 10 fields of view). (C) A confocal z-series through the nuclei of HeLa cells infected for 3 h with EPEC after treatment with leptomycin B and stained for nucleolin (green), actin (red) and DAPI (blue).
(0.17 MB PDF)

Figure S3 Effects of EPEC infection on nucleolar proteins and snoRNA. (A) Cellular location of prominent nucleolar proteins using N-terminal EGFP fusions and expressed in HeLa cells before and after a 3 h EPEC infection. Cells were co-stained with nucleolin antibodies (red). Arrow indicates nucleolin is the only EGFP fusion to enter the cytoplasm. (B) Immunofluorescence for the nucleolar markers fibrillarin (green) and BMS1 (red) before and after a 3 h EPEC infection in HeLa cells. (C) In situ hybridisation for U8 and U3 snoRNA after a 1 and 3 h infection with EPEC strains. (D) Quantification of cytoplasmic nucleolin in cells before and after transfection with EGFP-RPS5 (mean ± SE, 10 cells counted for each treatment).
(0.25 MB PDF)

Figure S4 Transfection of EspF and EspF variants into HeLa cells. (A) Magnification of Figure 5A and colocalisation (inset) of EspF-EGFP with nucleolin in transfected cells (B) Relative levels of expression of L16E-EspF-EGFP in transfected cells at different days post transfection measured by quantification of fluorescent signal (10 cells were randomly chosen each day from one of three separate experiments, mean ± SE shown) (C) Percentage of nucleolin negative cells after transfection with the indicated control plasmids (x-axis). Results represent the mean percentage ± SE of 15 randomly chosen transfected cells.(D) Representative image of a HeLa cell transfected with pL16E-*espF*delta21-74EGFP showing no nucleolar accumulation but with nuclear and cytoplasmic localisation. The right image shows the DAPI-stained nucleus from the cell with the nucleoli clearly evident (pseudo-coloured red).
(0.12 MB PDF)

Figure S5 Nucleolin or EspF levels in HeLa cells infected with non-EPEC strains. (A) Enlarged confocal image from Figure 6Aa showing actin pedestal formation on HeLa cells by *Citrobacter rodentium* (B) Representative confocal image showing EspF staining pattern in HeLa cells following *Citrobacter* infection (C) Quantification of EspF in from three Western blots of lysates from HeLa cells infected with the indicated bacterial species for the indicated time points. Data points represent relative densitometrical values (mean ± SEM). (D) Non-polarised TC-7 cells infected with *Salmonella typhimurium* SPI-2 mutant for 3 h induced extensive nucleolin redistribution into the cytoplasm. Image shows confocal section of cells stained for nucleolin (green), DAPI (blue) and actin (red) and revealed little nucleolin in the nucleus. (E) Magnification of red box in Figure 6Db showing nucleolin (green) recruitment around intracellular bacteria(blue). (F) HeLa cells infected for 8 h with *S. typhimurium* showing cytoplasmic nucleolin (green) sequestered around the intracellular bacteria (blue) with regions of the cytoplasm (arrow) displaying no or little nucleolin.
(0.17 MB PDF)

Protocol S1 Extended Protocols
(0.05 MB DOC)
