# Supplementary material for: The Enteropathogenic E. coli Effector EspF Targets and Disrupts the Nucleolus by a Process Regulated by Mitochondrial Dysfunction
Source: PLoS Pathog. 2010 Jun 24;6(6):e1000961. doi: 10.1371/journal.ppat.1000961 (PMC2891835; doi:10.1371/journal.ppat.1000961)
Supplement: Table S1 — Cell lines, Bacterial Strains, Plasmids, and Oligonucleotides (0.04 MB DOC) [file ppat.1000961.s001.doc]

| **Cell line** | **Description and Origin** | |
| --- | --- | --- |
| Caco-2 | A model for small intestinal enterocytes. Acquired from ATCC (No. HTB-37) | |
| TC-7 | A well characterised Caco-2 cell clone with a homogeneous and extensive brush border [38] | |
| HeLa | ATCC (No. CCL-2) Human cervical carcinoma cells | |
| **Strains** | **Description** | **Reference** |
| **Enteropathogenic *E. coli***  E2348/69  ∆*map*  ∆*tir*  ∆*eae* (CVD206)  ∆*espF*  ∆*espGorf3core* (goc)  ∆*espA*  **Enterohemorrhagi*c E.coli***  Wild type  ***Salmonella enteric* serovar *typhimuirum***  Wild type  ∆SPI-1 SLI344  ∆SPI-2 SLI344  ***Salmonella enteric* serovar *dublin***  Wild type  ***E.coli* RDEC-1**  Wild type  ***Citrobacter rodentium***  Wild type | Wild type EPEC 0127:H6  Deleted for *map*  Deleted for *tir*  Deleted for *eae* (Intimin)  Deleted for *espF*  Absence of *espG*, *orf3*, *map*, *tir*, *espH*, and chaperones *cesF, cesT*  Deleted for *espA*  0157:H7  SLI344  Defective SPI-1 TTSS  Defective SPI-2 TTSS | [39]  [11]  [9]  [40]  [41]  [17]  [42]  see [43]  Dr. Anjam Khan, University of Newcastle  Prof David Holden, Imperial College, London  The original Cantey and Blake strain  Provided by Dr Eric Oswald, Ecole Nationale Vétérinaire de Toulouse  Provided by Dr Eric Oswald, |
| **Plasmids** | **Description and Origin** | |
| pEGFP-N1, pEGFP-N3 | Clontech; for making C-terminal EGFP fusions | |
| pDsRED-MITO  pEGFP-MITO | Mitochondrial targeting sequence of cytochrome c oxidase fused to DsRED [37].This study. As above with the MTS cloned into pEGFPN1 | |
| pEGFP-NUCLEOLIN  pEGFP-B23  pEGFP-FIBRILLARIN  pEGFP-UBF1  pEGFP-RPS5  pEGFP-RPL9 | Full length genes were cloned into pEGFP-C1, generating an N-terminal EGFP fusion [21] | |
| p(L16E)EspF- EGFP  pEspF-EGFP | Full length genes cloned into pEGFP-N3 creating C-terminal EGFP fusions [16] | |
| p(L16E)EspF-EGFP deletion constructs:  Δ21-74  Δ101-184  Δ50-194 | This study | |
| pTCCP-EGFP | This study | |
| pEHEC-EspF-EGFP | This study | |
| pEGFP-EspF | An N-terminal EGFP fusion to full length EspF [44] | |
| pEspF(L16E)  pEspF | Fragment containing the full length EspF gene or L16E mutant cloned into pBR322 [16]. | |
| pSK-mapHA | [11] | |
| **Oligonucleotides** | **Sequence** | |
| PD1 EcoRI espF PS  PD2 BamHI espF NS  EspF 21 NS  EspF 50 NS  EspF 100 NS  EspF 74 PS  EspF 184 PS  EspF 194 PS | CGGAATTCATGCTTAATGGAATTAGTAACG  CGCGGATCCCGCCCTTTCTTCGATTGCTCAT CACAGATCTTGCGATACCTACAAGCTGC  CGAAGATCTAGAAAAGGGGAATGAAC  ATCAGATCTTGCCTGTGCAATGGGCGGTAA  ACTTCATTTACTAGATCTCGTCCGGCA  TCCAGATCTGGTGGTCTACCGCCCCTT  GTAAGCTTTAAGAGATCTCGTCAGGCA | |
