# Supplementary figures and images for: The Enteropathogenic E. coli Effector EspF Targets and Disrupts the Nucleolus by a Process Regulated by Mitochondrial Dysfunction
Source: PLoS Pathog. 2010 Jun 24;6(6):e1000961. doi: 10.1371/journal.ppat.1000961 (PMC2891835; doi:10.1371/journal.ppat.1000961)

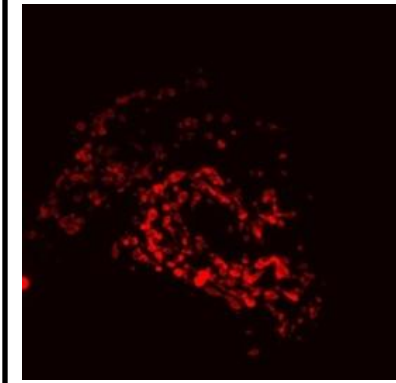

A

DsRED-MITO

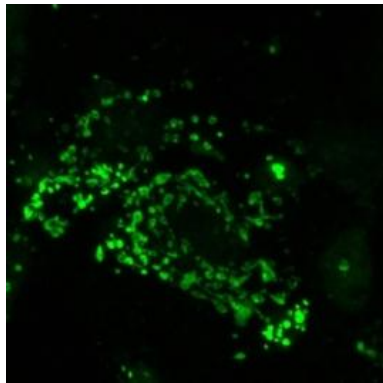

Anti-EspF

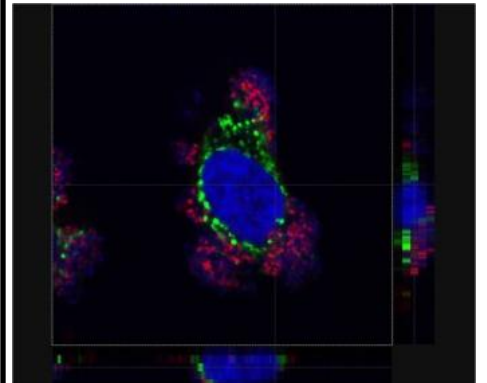

B

Actin/MapHA/DNA

Figure S1

Supplement: Figure S1 — EspF and Map staining in infected HeLa cells. (A) Colocalisation of DsRED-MITO (a mitochondrial marker, red) with EspF (green) in HeLa cells following a 60 min infection with EPEC. (B) Immunofluorescence using HA antibodies to detect MapHA (green) in HeLa cells after a 3 h infection with Δmap/pmapHA. Actin staining (red) shows pedestals on the cell surface; DNA (blue). (0.03 MB PDF) [file ppat.1000961.s002.pdf]

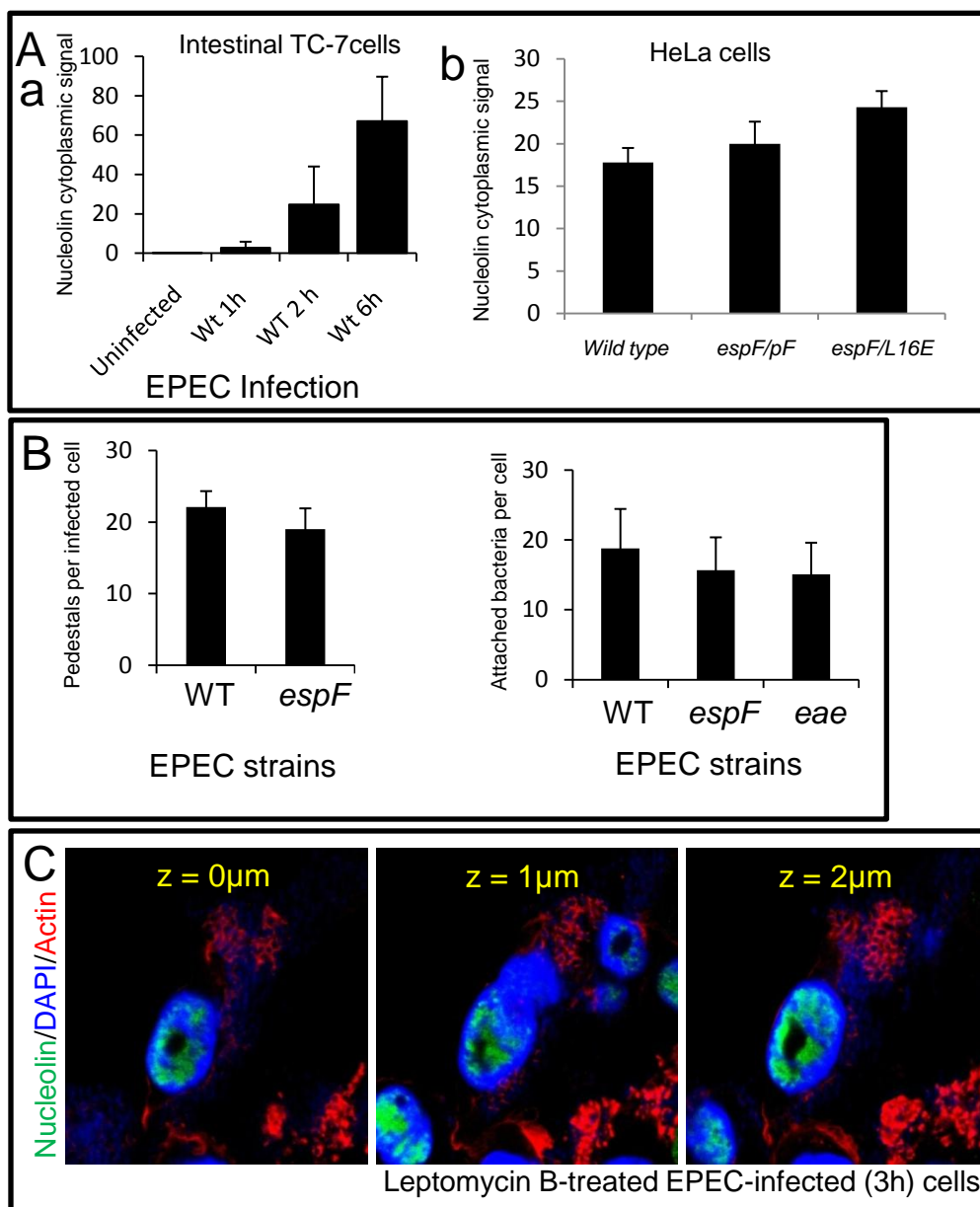

Figure S2

Supplement: Figure S2 — Nucleolin localisation and bacterial binding in host cells infected with EPEC. (A) (a) Quantification of the cytoplasmic nucleolin signal in EPEC infected TC-7 intestinal cells. Cytoplasmic nucleolin levels were counted over 6 fields of view (results show mean ± SE). (b) Quantification of the cytoplasmic nucleolin signal in HeLa cells infected with the indicated EPEC strains. (B) Left Graph: Number of pedestals per infected cell was not significantly different between WT and espF mutant after 40 min infection period (10 fields of view counted, results show mean ± SE). Right Graph: The number of bacteria attached to infected host cells after 40 min infection with indicated EPEC strains (results show mean ± SE, 10 fields of view). (C) A confocal z-series through the nuclei of HeLa cells infected for 3 h with EPEC after treatment with leptomycin B and stained for nucleolin (green), actin (red) and DAPI (blue). (0.17 MB PDF) [file ppat.1000961.s003.pdf]

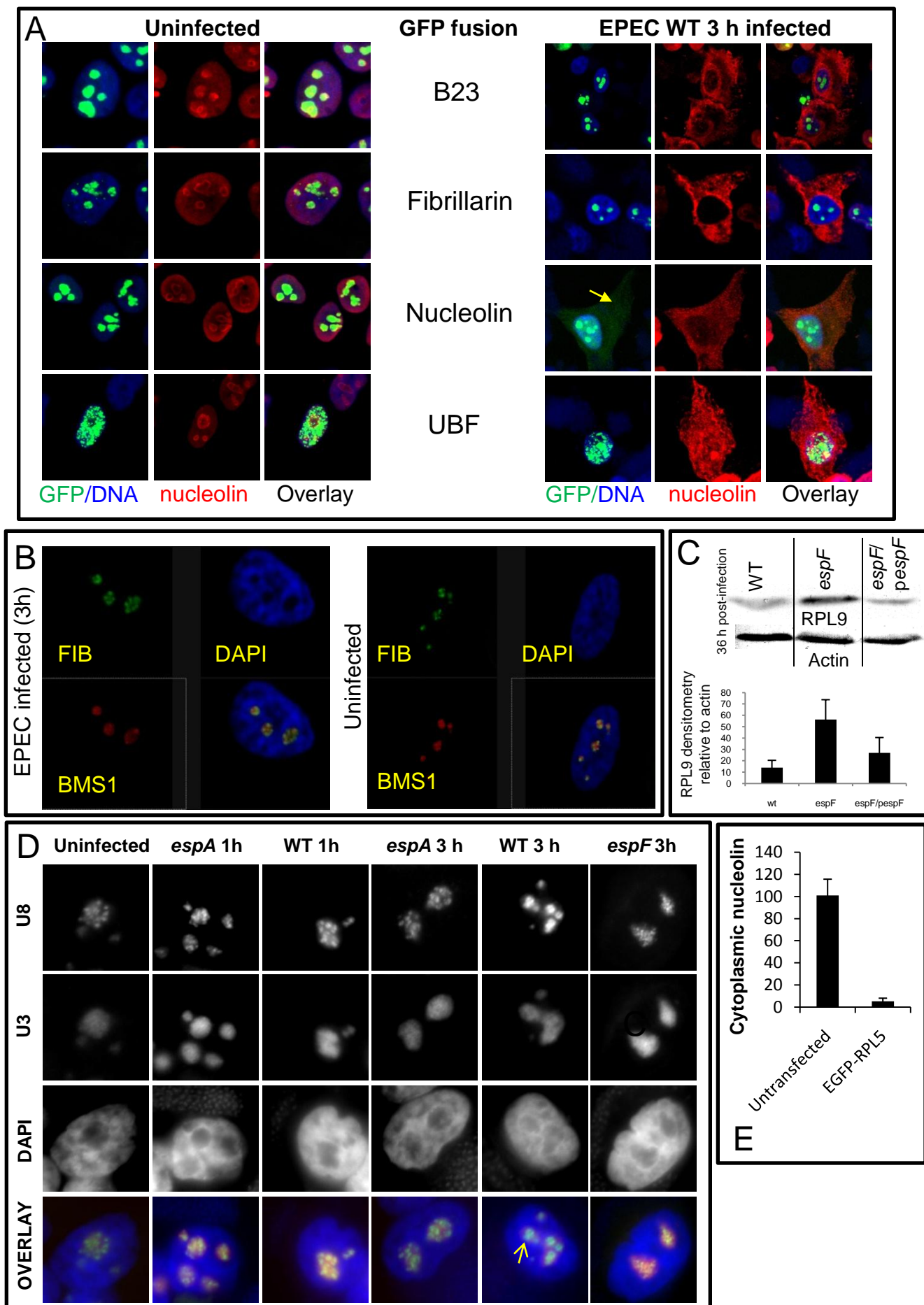

Figure S3

Supplement: Figure S3 — Effects of EPEC infection on nucleolar proteins and snoRNA. (A) Cellular location of prominent nucleolar proteins using N-terminal EGFP fusions and expressed in HeLa cells before and after a 3 h EPEC infection. Cells were co-stained with nucleolin antibodies (red). Arrow indicates nucleolin is the only EGFP fusion to enter the cytoplasm. (B) Immunofluorescence for the nucleolar markers fibrillarin (green) and BMS1 (red) before and after a 3 h EPEC infection in HeLa cells. (C) In situ hybridisation for U8 and U3 snoRNA after a 1 and 3 h infection with EPEC strains. (D) Quantification of cytoplasmic nucleolin in cells before and after transfection with EGFP-RPS5 (mean ± SE, 10 cells counted for each treatment). (0.25 MB PDF) [file ppat.1000961.s004.pdf]

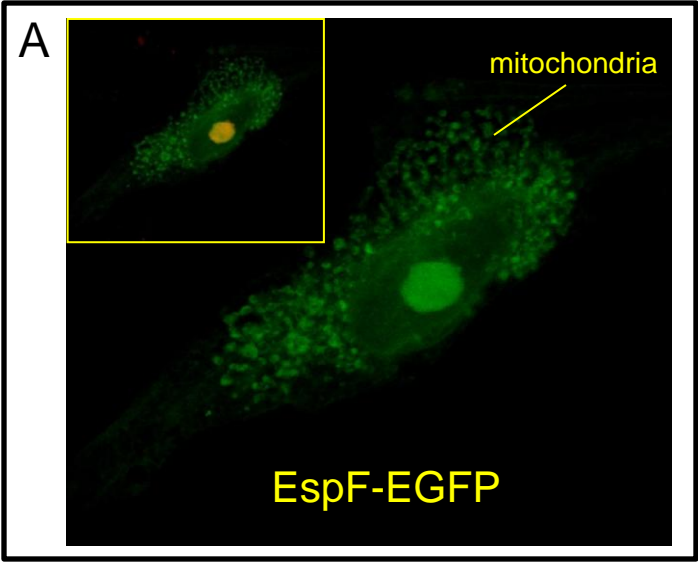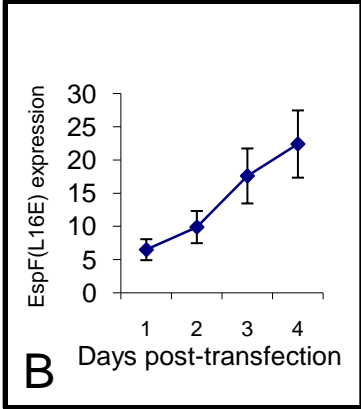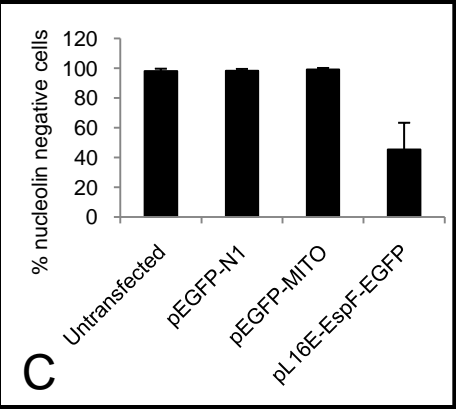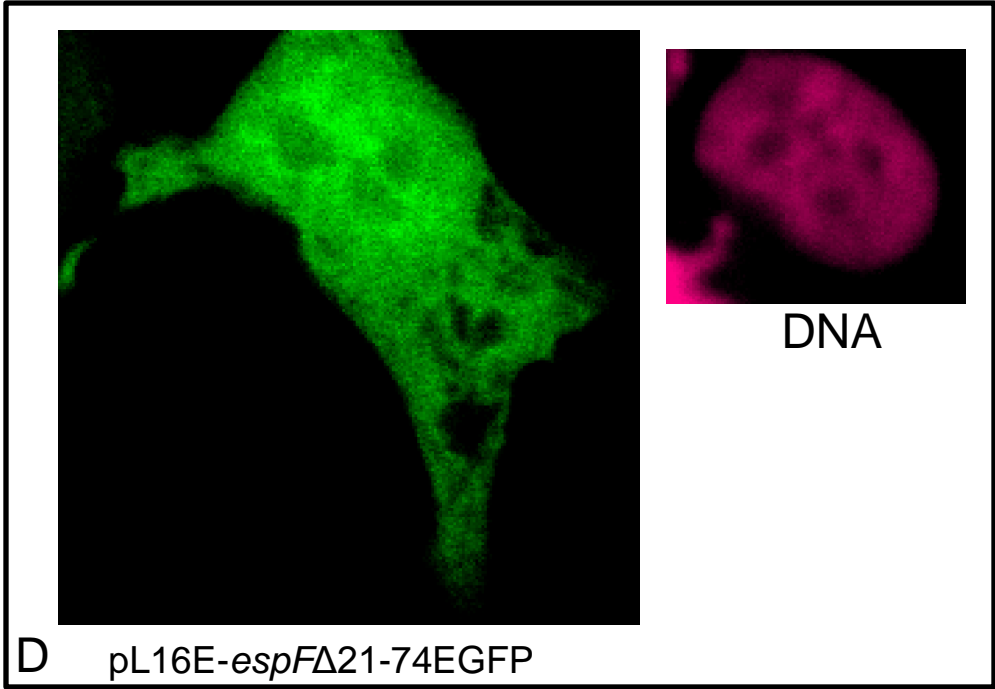

Figure S4

Supplement: Figure S4 — Transfection of EspF and EspF variants into HeLa cells. (A) Magnification of Figure 5A and colocalisation (inset) of EspF-EGFP with nucleolin in transfected cells (B) Relative levels of expression of L16E-EspF-EGFP in transfected cells at different days post transfection measured by quantification of fluorescent signal (10 cells were randomly chosen each day from one of three separate experiments, mean ± SE shown) (C) Percentage of nucleolin negative cells after transfection with the indicated control plasmids (x-axis). Results represent the mean percentage ± SE of 15 randomly chosen transfected cells.(D) Representative image of a HeLa cell transfected with pL16E-espFdelta21-74EGFP showing no nucleolar accumulation but with nuclear and cytoplasmic localisation. The right image shows the DAPI-stained nucleus from the cell with the nucleoli clearly evident (pseudo-coloured red). (0.12 MB PDF) [file ppat.1000961.s005.pdf]

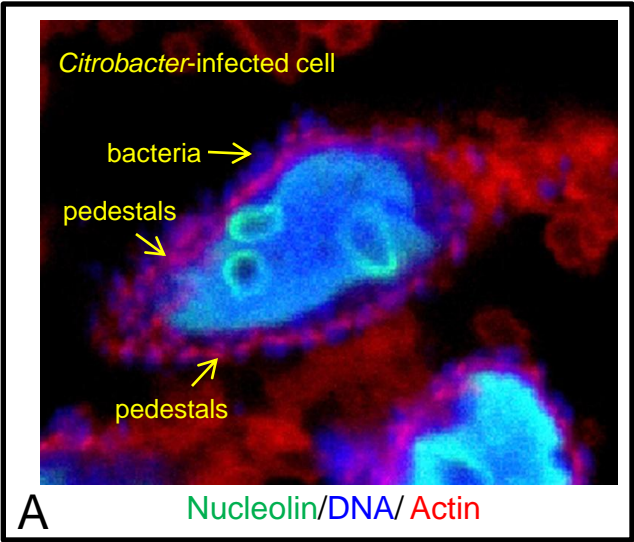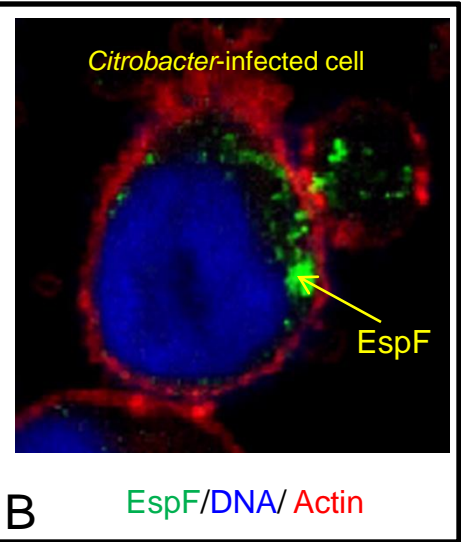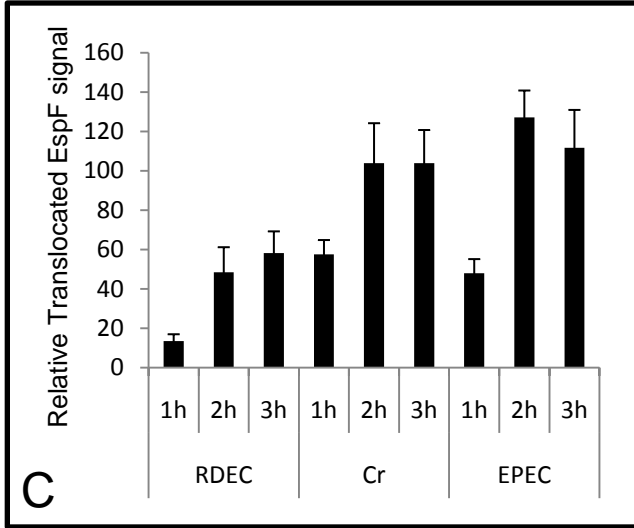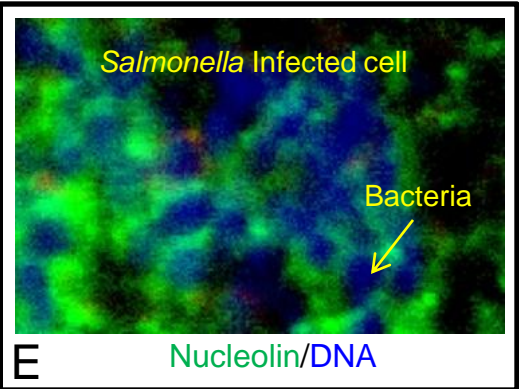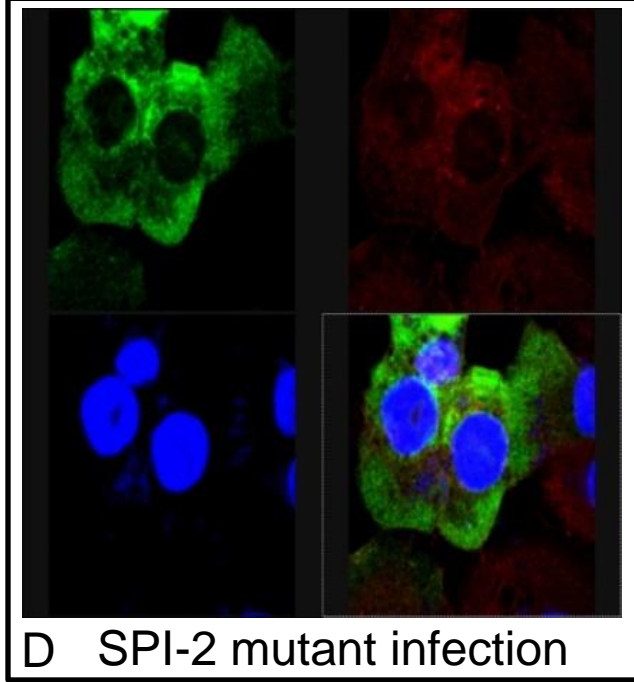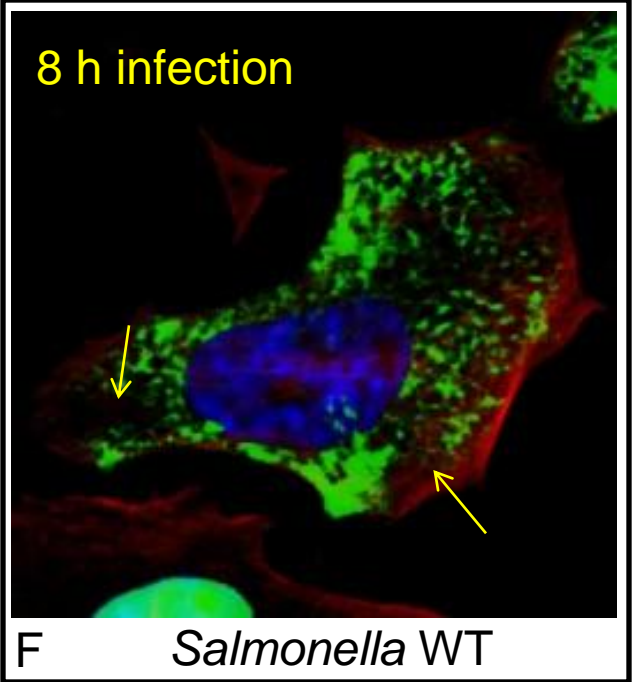

Figure S5

Supplement: Figure S5 — Nucleolin or EspF levels in HeLa cells infected with non-EPEC strains. (A) Enlarged confocal image from Figure 6Aa showing actin pedestal formation on HeLa cells by Citrobacter rodentium (B) Representative confocal image showing EspF staining pattern in HeLa cells following Citrobacter infection (C) Quantification of EspF in from three Western blots of lysates from HeLa cells infected with the indicated bacterial species for the indicated time points. Data points represent relative densitometrical values (mean ± SEM). (D) Non-polarised TC-7 cells infected with Salmonella typhimurium SPI-2 mutant for 3 h induced extensive nucleolin redistribution into the cytoplasm. Image shows confocal section of cells stained for nucleolin (green), DAPI (blue) and actin (red) and revealed little nucleolin in the nucleus. (E) Magnification of red box in Figure 6Db showing nucleolin (green) recruitment around intracellular bacteria(blue). (F) HeLa cells infected for 8 h with S. typhimurium showing cytoplasmic nucleolin (green) sequestered around the intracellular bacteria (blue) with regions of the cytoplasm (arrow) displaying no or little nucleolin. (0.17 MB PDF) [file ppat.1000961.s006.pdf]
