# Supplementary material for: The Enteropathogenic E. coli Effector EspF Targets and Disrupts the Nucleolus by a Process Regulated by Mitochondrial Dysfunction
Source: PLoS Pathog. 2010 Jun 24;6(6):e1000961. doi: 10.1371/journal.ppat.1000961 (PMC2891835; doi:10.1371/journal.ppat.1000961)
Supplement: Protocol S1 — Extended Protocols (0.05 MB DOC) [file ppat.1000961.s007.doc]

**Protocol S1**

**Bacterial Strains, Plasmids, and cell culture**

Bacterial strains, plasmids and oligonucleoltides used in this study are given in Table S1. Bacteria were cultured at 37°C on Luria-Bertani (LB) agar plates taken from glycerol stocks. Prior to infections, bacteria were grown at 37°C as standing cultures overnight in LB broth with appropriate antibiotics (nalidixic acid, kanamycin or carbenicillin 50, 25 and 100 µg/ml final concentration respectively). Mammalian cells used in this study were TC-7 (a Caco-2 subclone) and HeLa, obtained and described in Table S1. Cells were cultured as described previously [13, 36].

**Reagents used**

Reagents used in this study were leptomycin B (Calbiochem), TRITC-labelled phalloidin (Invitrogen), valinomycin (Sigma), Lipofectamine 2000 (Invitrogen) and Mowiol (Calbiochem). Antibodies were against nucleolin (Abcam), C23 (Santa Cruz Biotechnology), BMS1 and Fibrillarin (from the lab of Dr Nick Watkins, University of Newcastle), Actin (Sigma), EspF and Tir antibodies were obtained commercially from Dr Roger James (Department of Surgery, Leicester University), HA epitope tag (Covance), Alexa secondary mouse and rabbit antibodies 488 and 555 (Invitrogen), Alkaline phosphatase secondary antibodies (Zymed), Peroxidise-conjugated secondary antibodies (Jackson Laboratories).

**Infection assays**

HeLa cells were grown on glass coverslips at approximately 60-80% confluence prior to bacterial infection. Polarised cells were typically grown on membrane filters (0.4 µm pore size; Corning) for 10-15 days post-confluence prior to infection assays. To assess levels of ribosomal protein L9 in infected TC-7 cells, the cells were infected for 5 h with various EPEC strains and the bacteria were killed by exposure to 100 µg/mL gentamycin for 1 h. Cells were left for an additional 36 h, after which they were lysed with triton X-100 and processed for Western blot as described below. A double thymidine block was performed on non-polarised cells to synchronise the cell-cycle for nucleolin imaging by incubating cells in DMEM containing 2 mM thymidine for 19 h, removing the thymidine for 10 h and replenishing 2 mM thymidine for a further 17 h. After this time the thymidine was removed and the synchronised cells were used the following day(s). Prior to infections, all cell monolayers were washed twice in pre-warmed DMEM without supplements. Standing overnight bacterial cultures were diluted in DMEM (without supplements) to an OD_600_ of 0.03 and grown at 37°C in 5% CO_2_ for 3 h to pre-activate the type three secretion system. At this time, the OD_600_ was adjusted to 0.1 and 500µl of bacterial suspension was added to adherent cells. Infection times depended on bacterial species and experiment as given in the results section. For *Salmonella* infections, cultures prepared as above, were diluted 10 fold to an OD_600_ of 0.01 prior to addition to mammalian cells. Measurements of transepithelial electrical resistance were taken each hour using an EVOM epithelial voltohmeter (World Precision Instruments) as previously described [13]. Prior to some infections, cells were incubated in the presence of pharmacological agents for 2 h including valinomycin (1µM) or leptomycin B (5ng/mL).

**Indirect immunofluorescence (IF)**

Indirect IF was performed as described previously [13] with the following modifications. All secondary antibodies were conjugated to Alexa 488 or Alexa 543 (Invitrogen). Fixed and stained cells on coverslips or membrane filters were mounted with Mowiol reagent (Calbiochem) containing DAPI (2µg/ml) and allowed to set for 2h at room temperature. Slides were viewed either by confocal microscopy (as below) or with a Zeiss Axioscope phase contrast epifluorescence microscope and captured using a Hamamatsu C4742-95 camera with Openlab or Velocity software.

**Confocal and scanning microscopy and image analysis**

Confocal analysis was performed on a Leica TCS SP2UV confocal microscope. Fixed and stained cells on coverslips or membrane filters were visualised with a X63 objective lens and cross-sectioned along the ‘z-axis’ by making serial optical slices (parallel to the coverslip) from the top to the base of cells. Images were routinely deconvolved using Huygens professional deconvolution software using default parameters but with at least 50 iterations. Maximal confocal projections (entire reconstructed ‘z-stack’ along the z-axis) or single z-slices are indicated in the results section. Fluorescence intensity was determined using Leica quantification software or Image J (NIH) and presented as arbitrary fluorescence values based on the mean numbers of pixels for each channel. Total fluorescence from individual cells was determined by capturing the cell as a region of interest (ROI) using confocal software. The nuclear/nucleolar signal was measured by making an ROI around these cellular structures while the cytoplasmic signal was determined by subtracting the nuclear signal from the total cell fluorescence. Routinely, negative control slides were used to set base parameters for each series of slides, which was maintained during visualisation, ensuring the detected signal was specific to the fluorophore being examined.

**Cell fractionation and Western blot analysis**

Caco-2 cells were infected for indicated times and fractionated according to [13] with some modifications. Briefly, on ice and with 1x protease inhibitor cocktail (Sigma) in all buffers, infected cells were exposed to 0.2% (v/v) saponin (Calbiochem), sedimented (12,000 g) and the ‘cytoplasmic’ supernatant collected. The membranous pellet was washed in PBS and lysed with 1% (v/v) Triton X-100 (Sigma), sedimented as before and the ‘membrane’ fraction was collected. The ‘insoluble’ pellet, which contains the nucleus, bacteria and cytoskeleton, was resuspended in PBS. Protein concentrations of all samples were determined by Bradford’s reagent and diluted to the same protein concentration in Laemmlli sample buffer prior to boiling and loading onto an SDS-polyacrylamide gel. The nuclear extraction kit was performed according to the manufacturer’s instructions (Sigma). Western blot analysis was performed as described elsewhere [13] and antibody detection was by chemiluminescence (Pierce) or alkaline phosphatase (Promega)

**Transfection**

Transfection of all cell types was performed using Lipofectamine 2000 (Invitrogen) according to the manufacturer’s instructions. Briefly, cells grown on coverslips (50-80% confluence) were transfected with 1:3 ratio of DNA:lipofectamine, left for 2 h at 37°C in 5% CO_2_, washed in DMEM and incubated for up to 7 days post-transfection (indicated in results section). Typically, cells were visualised after 48 h post-transfection unless otherwise stated. Endotoxin-free plasmid DNA, purified up to 3 mg/mL (Qiagen midiPrep kit) was used for all transfections.

**Generation of plasmids and EspF deletion constructs**

Standard cloning techniques were used to generate the plasmids (Table S1) in this study. Briefly, pEGFPN1-MITO was generated by digesting pDsRED-MITO [37] with NdeI/HindIII to give a fragment harbouring the CMV promoter and the mitochondrial targeting sequence. This fragment was subcloned directly between the NdeI/HindIII sites of pEGFP-N1, generating an in-frame fusion of the MTS with EGFP. EspF(L16E) was cloned into pBR322 [16] and using inverse PCR with oligonucleotides shown in Table S1, the region to be deleted was exchanged for a 6-bp insert containing the BglII restriction site. To generate EGFP fusions, the deletion constructs were amplified by PCR using the oligonucleotides PD1 and PD2 given in Table S1 and cloned into pEGFP-N3, creating in-frame fusions with EGFP. All newly-made constructs were verified by DNA sequencing (GATC-Biotech).

**Fluorescence in situ hybridisation**

HeLa cells grown at 70% confluency were infected for 1 or 3 h, washed three times in phosphate buffered saline (PBS) and fixed in 2.5% PBS/paraformaldehyde. In situ hybridisation was then performed using oligonucleotides according to [20]. Oligonucleotides were conjugated to fluorophores (Invitrogen) using N-hydroxysuccinimide (Amersham) as described by the manufacture. Images were acquired by epifluorescent microscopy at x100 as described above.

**Statistical analysis and Bioinformatics**

In all cases, unless otherwise stated, experiments were repeated independently at least 3 times. Presented graphs represent the mean ± SEM and where confocal microscopy was used for quantification, results represent at least 50 cells for each experiment over 3-5 randomly chosen fields of view. Where necessary, comparison of means was performed using the non-parametric Mann-Whitney U test with p values less than 0.05 taken as a significant difference. Bioinformatic analysis of the EspF protein was performed using BLASTP, BLAST PSI, PredictNLS (http://cubic.bioc.columbia.edu/cgi/var/nair/resonline.pl) and MITOPROT (www.expasy.org).
